# Prognostic relevance of autophagy-related markers LC3, p62/sequestosome 1, Beclin-1 and ULK1 in colorectal cancer patients with respect to KRAS mutational status

**DOI:** 10.1186/s12957-016-0946-x

**Published:** 2016-07-22

**Authors:** Klaus Juergen Schmitz, Ceflije Ademi, Stefanie Bertram, Kurt Werner Schmid, Hideo Andreas Baba

**Affiliations:** Institute of Pathology, Mühlenstrasse 31, 45659 Recklinghausen, Germany; Institute of Pathology, University Hospital Essen, University of Duisburg-Essen, Hufelandstrasse 55, Essen, 45147 Germany; Department of Senology, Prosper Hospital Recklinghausen, Mühlenstrasse 27, 45659 Recklinghausen, Germany

**Keywords:** Colorectal cancer, Autophagy, p62, Beclin-1, LC3, ULK1

## Abstract

**Background:**

Autophagy is a cellular pathway that regulates transportation of cytoplasmic macromolecules and organelles to lysosomes for degradation. Autophagy is involved in both tumorigenesis and tumour suppression. Here we investigated the potential prognostic value of the autophagy-related proteins Beclin-1, p62, LC3 and uncoordinated (UNC) 51-like kinase 1 (ULK1) in a cohort of colorectal cancer (CRC) specimens.

**Methods:**

In this study, we analysed the immunoexpression of the autophagy-related proteins p62, LC3, Beclin-1 and ULK1 in 127 CRC patients with known KRAS mutational status and detailed clinical follow-up.

**Results:**

Survival analysis of p62 staining showed a significant correlation of cytoplasmic (not nuclear) p62 expression with a favourable tumour-specific overall survival (OS). The prognostic power of cytoplasmic p62 was found in the KRAS-mutated subgroup but was lost in the KRAS wildtype subgroup. Survival analysis of Beclin-1 staining did not show an association with OS in the complete cohort. LC3 overexpression demonstrated a slight, though not significant, association with decreased OS. Upon stratifying cases by KRAS mutational status, nuclear (not cytoplasmic) Beclin-1 staining was associated with a significantly decreased OS in the KRAS-mutated subgroup but not in the KRAS wildtype CRCs. In addition, LC3 overexpression was significantly associated with decreased OS in the KRAS-mutated CRC subgroup. ULK1 expression was not correlated to survival.

**Conclusions:**

Immunohistochemical analyses of LC3, p62 and Beclin-1 may constitute promising novel prognostic markers in CRC, especially in KRAS-mutated CRCs. This strategy might help in identifying high-risk patients who would benefit from autophagy-related anticancer drugs.

**Electronic supplementary material:**

The online version of this article (doi:10.1186/s12957-016-0946-x) contains supplementary material, which is available to authorized users.

## Background

Macroautophagy, herein referred to as autophagy, is a process that allows cells to deliver intracellular proteins, lipids and organelles to lysosomes where degradation can take place [1]. After autophagic degradation, the intracellular material is removed from the lysosomal compartment and recycling occurs in the cytoplasm [2]. The impact of autophagy on cellular physiology varies and depends upon the circumstances of the affected cell. The process of autophagy is also involved in tumorigenesis and can be both tumour promoting as well as tumour suppressing. Tumour-suppressive effects are achieved by degradation of oncogenic protein substrates, toxic proteins and defective organelles. However, autophagy-related intracellular recycling of substrates necessary for mitochondrial activity exerts a tumour-promoting effect in cancer cells. Autophagy can also function as a cancer cell survival pathway. Several studies have shown that autophagy is upregulated in RAS-driven cancers [3] and is especially induced in cancer areas with hypoxic conditions, where it supports tumour cell survival [4].

LC3, p62, Beclin-1 and uncoordinated (UNC) 51-like kinase 1 (ULK1) are central autophagy-related proteins involved in the autophagy flux. LC3 (microtubule-associated protein 1 light chain 3) is a well-established marker of autophagy activity in cancer cells [5]. LC3 is a mammalian homolog of the yeast ATG8 protein, a ubiquitin-like protein that becomes lipidated and tightly associated with autophagosomal membranes [6]. The hallmark of autophagosome formation is characterized by the insertion of LC3 II (isoform II of light chain) within the inner and outer layers of the vesicle. Measurement of LC3 expression by immunohistochemistry is a frequently used method to reliably quantify autophagosome formation [7].

p62 is an adaptor protein with several binding motifs and functions in assembling protein complexes [8]. p62 is involved in several important signalling pathways such as the NF-kB pathway [9]. In addition, p62 functions in the regulation of apoptosis via activation of polyubiquitinated caspase 8 [10] and influences the oxidative stress response, which is regulated by the Keap1-Nrf2 system. Under oxidative stress, p62 binds to Keap, which in turn blocks Nrf2 degradation, leading to increased expression of cytoprotective Nrf2 target genes.

Beclin-1 is an autophagy-specific protein that regulates autophagosome formation [11]. Until recently, the *BECN1* gene that encodes the Beclin-1 protein was proposed to function as a tumour suppressor gene. However, current studies have revealed no evidence for *BECN1* mutation or loss in cancers other than breast and ovarian cancers [12]. The allelic loss of *BECN1* in breast and ovarian cancer seems to not be a driver mutation, but a passenger mutation along with BRCA1 mutation due to the proximity of *BECN1* to *BRCA1*. Data from the Cancer Genome Atlas Research Network, accessible via the cBioPortal for Cancer Genomics, show that only 2.5 % of colorectal cancers (CRCs) show genetic alterations of the *BECN1* gene [13, 14]. According to the cBIOPortal data, the p62 gene is altered in 1.5 % of CRC and the LC3 gene is altered in up to 14 %.

Beclin-1 interacts with several binding partners and can both induce and suppress the autophagy pathway. Interaction with PI3K can lead to upregulation of autophagy while interaction with Bcl-2 can result in inhibition of autophagy [15]. While Beclin-1 was thought to localize mainly in the cytosolic department, several studies demonstrated both nuclear and cytoplasmic localization of Beclin-1. Indeed, in a study of a cohort of CRCs, nearly half of the cancers exhibited a significant nuclear Beclin-1 staining pattern [16].

ULK1 is a serine/threonine kinase which is essential for autophagy and is involved in early autophagosome formation. Under nutrient deprivation, ULK1 is activated by the activated AMP-activated protein kinase (AMPK) and induces initiation of autophagy [17].

In vitro studies demonstrated that treatment of tumour cell lines with autophagy inhibitors such as chloroquine increases chemotherapy-induced cell death, thus establishing autophagy as a promising novel therapeutic target [18–20]. Whether key autophagy proteins may predict chemoresistance in CRC is not known. Here we investigated the potential prognostic value of the autophagy-related proteins Beclin-1, p62, LC3 and ULK1 in a cohort of CRC specimens with a focus on patients with an unfavourable outcome due to UICC stage III/IV CRC treated with chemotherapy and/or KRAS-mutated CRCs.

## Methods

### Patients

This study comprised 127 consecutive CRC patients (66 males, 61 females) who underwent surgery from 1996 to 1998 according to the recommendations of the German Society of Surgery and who did not receive any neoadjuvant treatment. Total/partial mesorectal excision was not performed at this time. Detailed and complete clinical records and follow-up data were available in all cases. The minimum follow-up period for patients still alive was 60 months. Surgical specimens were fixed in formalin and routinely processed. Tumours were classified according to the TNM System (6th edition). Patients with UICC stages III and IV CRC received a standardized 5-fluorouracil (5-FU)-based chemotherapy in the adjuvant setting. According to the recommendations of the German Surgical Oncology Working group (CAO), patients with stage I and II colorectal carcinoma were not advised to any adjuvant treatment. Approximately 63 % of the tumours were located at the colon and 37 % were located at the rectum. Detailed information on grading, lymph node status, T stage and distant metastasis for all cases was available. Informed consent was obtained from every patient, and the study protocol conforms to the ethical guidelines of the 1975 Declaration of Helsinki.

### Tissue microarray construction

Multi-tissue blocks of previous studies [21] were used for the present immunohistochemical analysis. The most representative and vital tumour area was selected and marked on H&E (haematoxylin and eosin)-stained slides. There was no restriction to the tumour periphery or tumour centre. For the construction, we used a manual tissue-array instrument (Beecher Instruments, Silver Spring, MD, USA). At least three and a maximal six 0.6-mm-thick tissue cores were taken from each tumour. Each block contained normal mucosa from resection margins of diverticulosis specimen and cores with liver tissue for tissue microarray (TMA) orientation.

### Immunohistochemistry of TMA

Immunohistochemistry of p62, LC3, Beclin-1 and ULK1 was performed on 5-μm-thick paraffin sections. Antigen retrieval was carried out with a hot water bath at pH 9.0 (p62) for 20 min or pH 6.0 (Beclin-1) for 30 min in a hot water bath (95 °C). Sections were incubated with p62 antibodies (1:1000 dilution) or Beclin-1 antibodies (1:400) for 30 min at room temperature. Antibody retrieval for LC3B was carried out with hot water bath (95 °C) for 20 min at pH 9.0. Detection was performed with the Zytomed Polymer Kit (Zytomed Systems, Berlin, Germany). The monoclonal p62 antibody (clone D3, sc-28359) purchased from Santa Cruz Biotechnology (Heidelberg, Germany) is raised against amino acids 151–440 of SQMSTM1/p62 of human origin. The rabbit polyclonal Beclin-1 antibody (ab55878) was purchased from Abcam (Cambridge, UK). The LC3B rabbit polyclonal antibody was purchased from Cell Signalling Technology (Danvers, MA, USA) and was raised against the clone D11. Antigen retrieval for ULK1 was carried with a hot water bath (98 °C) at pH 9.0. Sections were incubated with the monoclonal ULK1 antibody (1:400) for 15 min and detection was performed with Envision™. The ULK1 antibody was purchased from Abcam, Cambridge, UK. In order the check the specificity of the used antibodies (Beclin-1, p62, LC3B), we conducted additional cell culture experiments and were able to demonstrate specificity of the antibodies after treatment with chloroquine. We performed cell culture experiments with HepG2 cells treated with chloroquine in a final concentration of 50 μmol for 0 and 24 h. The enclosed figures show no or weak immunoreactivity for LC3, Beclin-1 and p62 but strong reactivity after 24 h treatment (see Additional files 1 and 2: Fig. S1 and Table S1). This indicates at least in cell culture specificity for the target antigen.

### KRAS mutation analysis

Mutational analysis was previously performed by our group [21]. In short, exon 1 of KRAS, harbouring codons 12 and 13, was amplified by polymerase chain reaction (PCR) followed by direct sequencing.

### Statistical analysis

Semiquantitative ULK1, p62 and Beclin-1 staining were assessed by two of the authors (CA, KJS) and LC3 staining by two of the authors (CA, KJS) in a blind-trial fashion without knowledge of the clinical outcome. In cases with striking discordance, slides were re-evaluated by KJS and a final decision was made. All data were statistically analysed using SPSS Statistics Version 20. Relationships between ordinal parameters were investigated using two-tailed chi-square analysis. The Kaplan-Meier method was used to estimate overall survival (OS), and any differences in the survival curves were compared by log-rank test. For multivariate analysis, Cox regression was used. For both tests, a *p* value of 0.05 or less was considered to be of statistical significance. Overall, 95 % confidence intervals were used throughout this study.

## Results

### Immunohistochemical analysis of LC3, p62, Beclin-1 and ULK1

**Fig. 1:**
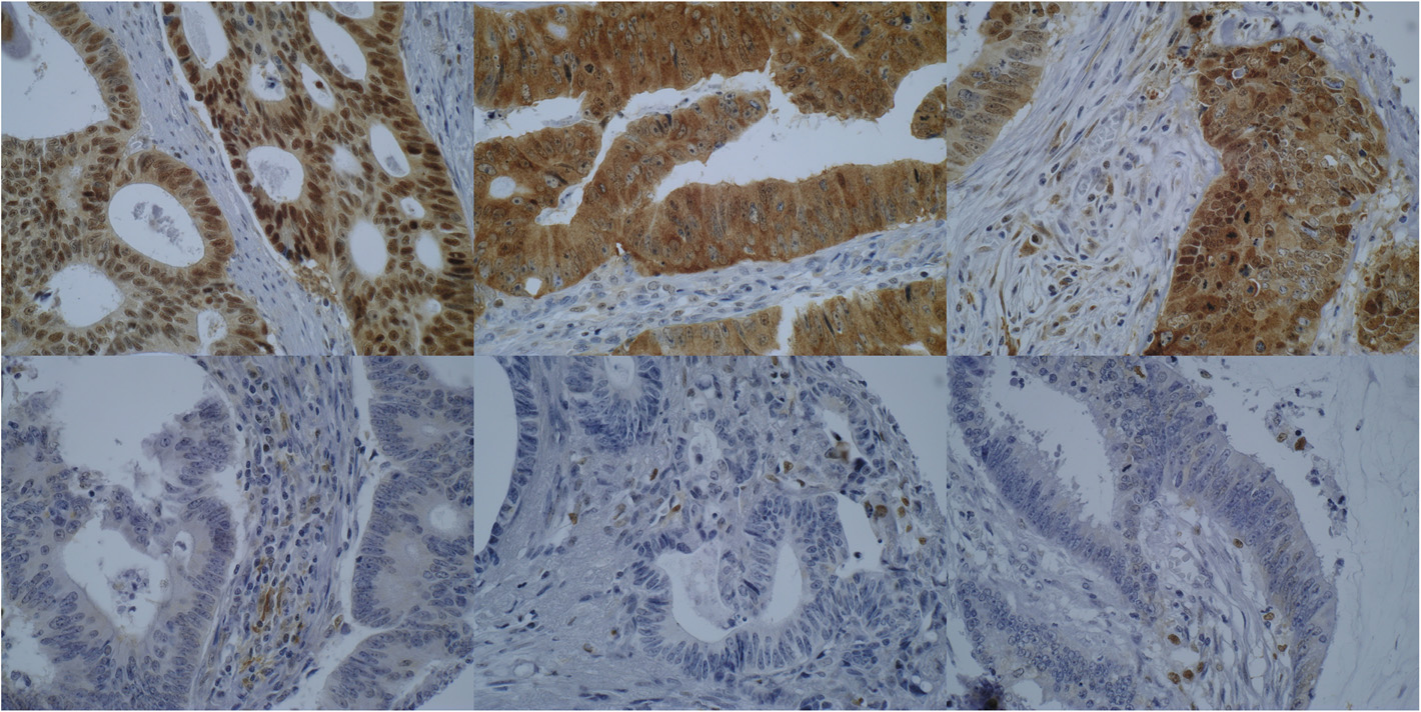
p62 immunohistochemistry. Examples of p62 immunostaining in colorectal cancers. The upper row shows three tumours with strong cytoplasmic immunostaining and the lower row demonstrates three tumours with negative p62 staining (×400 magnification). Note the positively stained of stromal cells/macrophages, which can be used as internal controls

**Fig. 2:**
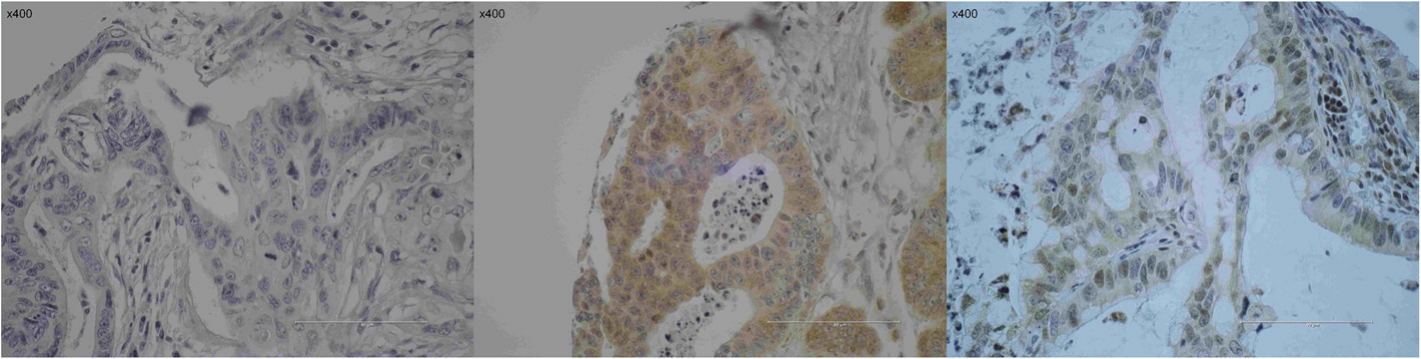
Beclin-1 immunhistochemistry. Examples of Beclin-1 immunostaining in colorectal cancers. On the *left*, there is a cancer without any Beclin-1 immunostaining. In the *middle*, there is a cancer with prominent cytoplasmic Beclin-1 immunostaining whereas on the *right* a colorectal cancer exhibits a mainly nuclear Beclin-1 expression (×400 magnification)

**Fig. 3:**
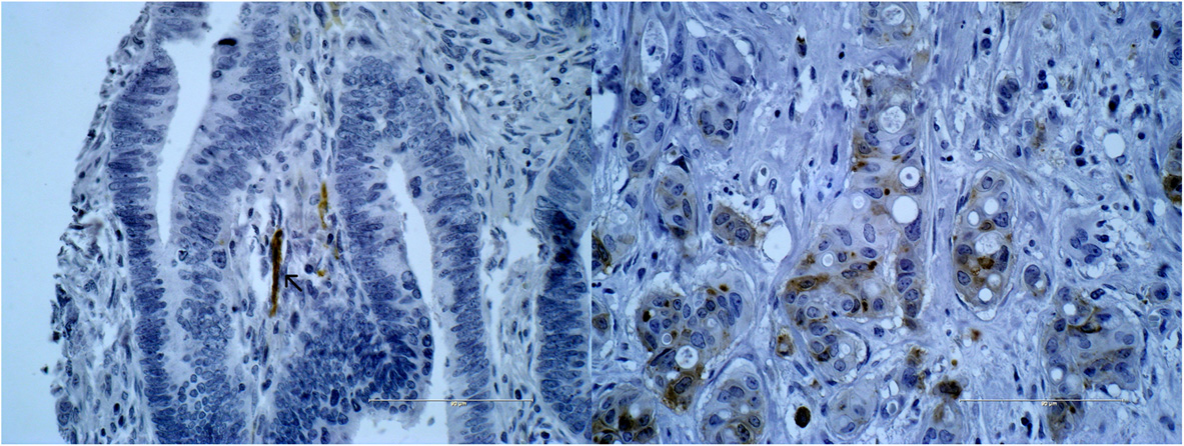
LC3 immunohistochemistry. On the *left side*, there is a cancer without any dot-like LC3 immunostaining. Notice the positively stained nerve, which can be considered as a positive internal control, since nerve fibers are constantly LC3 positive. On the *right side*, there is a CRC with a strong and characteristic dot-like LC3 immunostaining. A diffuse cytoplasmic staining was not noticed (×400 magnification)

**Fig. 4:**
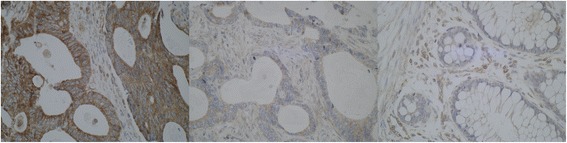
ULK1 immunhistochemistry. On the *left*, there is a picture of a cancer with strong cytoplasmic immunostaining. In the *middle*, there is a cancer with barely seen faint ULK1 staining, classified as negative. On the *right side*, missing staining in normal mucosa is demonstrated. Note the positivity in small blood vessels as an internal control (×400 magnification)

p62 and Beclin-1 immunohistochemistry of the tumour tissue revealed varying nuclear and cytoplasmic immunostaining. p62 and Beclin-1 immunohistochemistry demonstrated diffuse cytoplasmic staining and occasionally dot-like staining in the tumour specimens. Frequent detection of dot-like p62 and Beclin-1 staining was obstructed by the overwhelming presence of a diffuse staining pattern. Adjacent liver tissue on the same slide exhibited a dot-like p62 staining pattern. LC3 immunostaining showed a typical cytoplasmic dot-like staining and no diffuse cytoplasmic staining was observed. ULK1 staining showed a solely cytoplasmic and membranous staining pattern. Representative staining examples of p62, Beclin-1, LC3 and ULK1 are shown in Figs. 1, 2, 3 and 4.

**Fig. 5:**
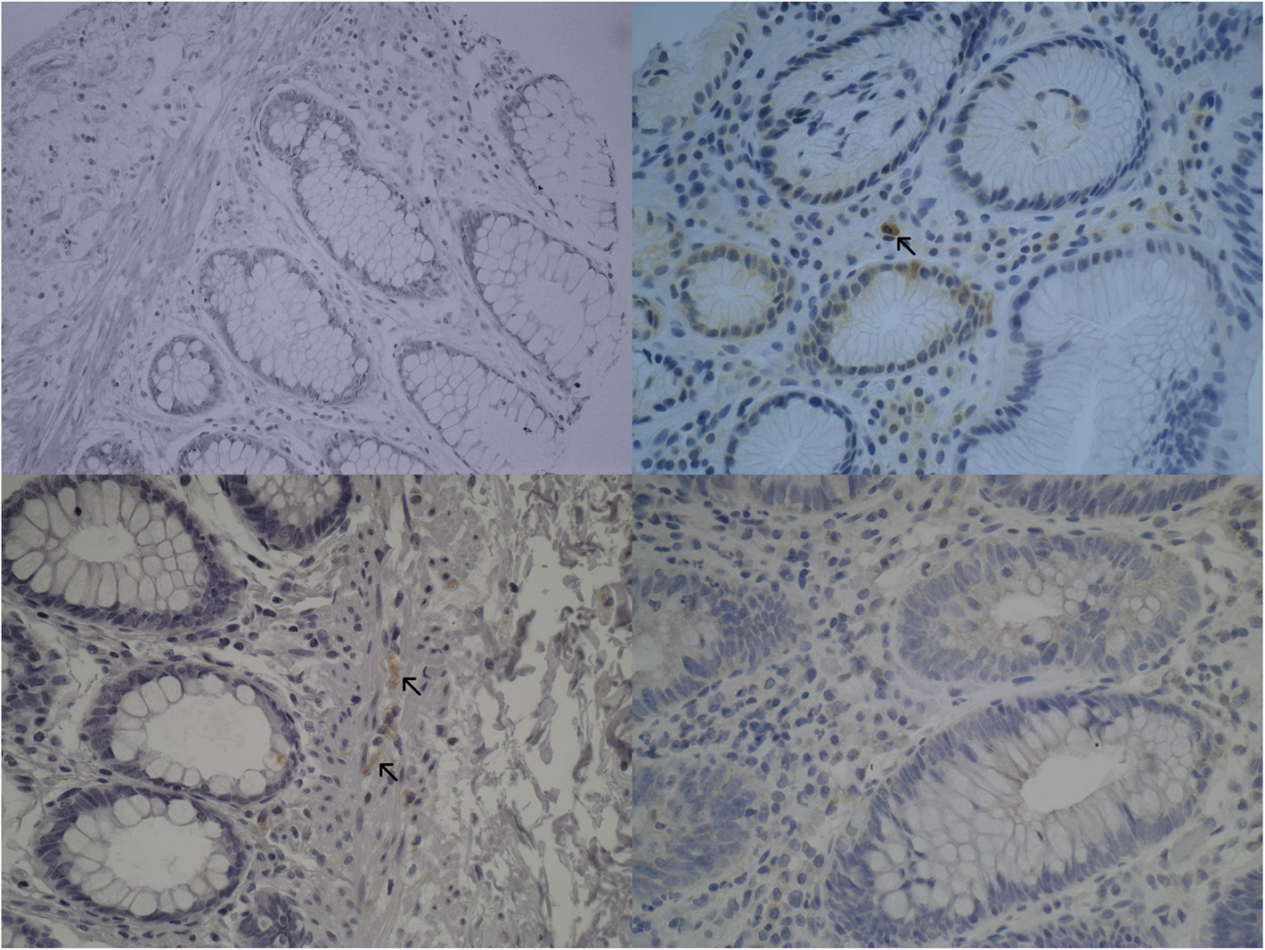
Autophagy-related proteins in normal colonic epithelial tissue. In the *upper left*, the picture shows normal crypts lacking Beclin-1 immunoexpression. In the *upper right*, negative p62 staining can be seen in normal colonic crypts. Notice the positive staining in macrophages. The *lower left* picture demonstrates lack of LC3B expression in crypts. Notice the moderate LC3B positivity in nerve cells. The *lower right* light micrograph shows a lack of ULK1 staining in normal crypts (×400 magnification)

Normal colon mucosal tissue from diverticulosis specimen was embedded in every single multi-tissue tumour block. Non-tumorous colonic epithelium lacked p62 and LC3B immunostaining. Macrophages and other stromal cells exhibited a weak to moderate p62 positivity. Neuronal cells demonstrated a moderate to strong LC3B immunoexpression. A missing to weak Beclin-1 immunostaining was noticed in normal mucosal epithelial tissue whereas normal colonic epithelium lacked any ULK1 immunostaining. A weak to moderate ULK1 staining of blood vessel walls can be used as internal ULK1 staining control. Staining results of non-tumorous colonic mucosa are shown in Fig. 5.

We next performed separate analyses of the different autophagy markers with respect to intensity and staining localization.

### Cytoplasmic p62 immunostaining

We classified tumour cytoplasmic staining as positive (+1) if one or more cores exhibited positive cytoplasmic p62 immunostaining regardless of staining intensity, or negative (0). Data for cytoplasmic p62 staining was available in 126 cases; in one case, all cores were lost from the slide. A total of 15 (11.9 %) tumours were classified as cytoplasmic p62 negative and 111 (88.1 %) were classified as cytoplasmic p62 positive. Statistical analysis revealed a trend towards cytoplasmic p62 negativity in patients with lymphonodal metastasis (*p* = 0.072; Table 1). Statistical chi-square analysis did not show correlations between p62/sequestosome1 and histological grading, tumour stage or distant metastasis.

**Table 1:** Immunostaining for cytoplasmic p62, nuclear Beclin-1 and LC3 expression

	Cytoplasmic p62 expression			Nuclear Beclin-1 expression			LC3 expression		
	Negative	Positive	*p* value	Negative	Positive	*p* value	Negative	Positive	*p* value
Histological grading			0.920			0.746			0.016
1	3 (10 %)	27 (90 %)		15 (53.6 %)	13 (46.4 %)		25 (83.3 %)	5 (16.7 %)	
2	9 (12.6 %)	61 (87.1 %)		37 (53.6 %)	32 (46.4 %)		50 (74.6 %)	17 (25.4 %)	
3	3 (11.5 %)	23 (88.5 %)		12 (63.2 %)	7 (36.8 %)		13 (50 %)	13 (50 %)	
Tumour stage			0.491			0.373			0.124
pT1	0 (0 %)	3 (100 %)		1 (33.3 %)	2 (66.6 %)		2 (66.7 %)	1 (33.3 %)	
pT2	0 (0 %)	12 (100 %)		4 (36.4 %)	7 (63.6 %)		10 (83.3 %)	2 (16.7 %)	
pT3	11 (14.3 %)	66 (85.7 %)		43 (60.6 %)	28 (48.3 %)		57 (76 %)	18 (24 %)	
pT4	4 (12.5 %)	28 (87.5 %)		15 (51.7 %)	14 (48.3 %)		17 (54.8 %)	14 (45.2 %)	
Lymph node metastases status			0.072			0.500			0.272
pN0	4 (6.9 %)	54 (93.1 %)		31 (55.4 %)	25 (44.6 %)		42 (75 %)	14 (25 %)	
pN+	11 (17.2 %)	53 (82.8 %)		30 (53.6 %)	26 (46.4 %)		43 (68.3 %)	20 (31.7 %)	
Distant metastasis			0.189			0.118			0.027
pM0	9 (9.6 %)	85 (90.4 %)		52 (57.8 %)	38 (42.2 %)		71 (77.2 %)	21 (22.8 %)	
pM+	5 (17.9 %)	23 (82.1 %)		9 (40.9 %)	12 (59.1 %)		15 (55.6 %)	12 (44.4 %)	
KRAS status			0.607			0.297			0.242
KRAS wildtype	11 (12.2 %)	79 (87.8 %)		46 (57.5 %)	34 (42.5 %)		66 (74.2 %)	23 (25.8 %)	
KRAS mutated	4 (11.8 %)	30 (88.2 %)		17 (50 %)	17 (50 %)		21 (65.6 %)	11 (34.4 %)	

### Nuclear p62 immunostaining

Data for nuclear p62 immunostaining was available for 125 patients. p62 nuclear staining was classified as negative (no nuclear or weak nuclear staining), low (moderate nuclear staining) or high (strong nuclear staining), independent of the amount of stained nuclei. A total of 45 (35.4 %) cases were classified as negative nuclear p62, 61 (48 %) as low nuclear p62 and 19 (15 %) as high nuclear expression. Chi-square analysis revealed no association of nuclear p62 staining with clinicopathological parameters. There was no association with OS (Kaplan-Meier analysis).

### Cytoplasmic Beclin-1 immunostaining

We classified Beclin-1 cytoplasmic staining of tumours as positive (+) if one or more cores exhibited a moderate or strong cytoplasmic Beclin-1 immunostaining regardless of amount of stained tumour cells or negative (0). Data for cytoplasmic Beclin-1 staining was available for 116 cases. Cores were lost on the slides for 11 cases. A total of 65 (56 %) were classified as negative and 51 (44 %) as positive. Statistical analysis showed no correlation with the clinicopathological parameters (histological grading, tumour stage, lymph node metastases status, distant metastases, or KRAS mutational status).

### Nuclear Beclin-1 immunostaining

Beclin-1 nuclear staining was classified as negative (0) and positive (+). Tumours missing nuclear Beclin-1 immunostaining or showing a weak nuclear Beclin-1 staining were classified as negative (0). Tumours showing a moderate or strong nuclear immunostaining (regardless of the amount of stained tumour cell nuclei) were classified as positive (+). Among the 116 total cases, 64 (55.2 %) were classified as negative and 52 (44.8 %) as positive. No statistical correlation was detected between nuclear Beclin-1 expression and the clinicopathological data (chi-square analysis).

### LC3 immunostaining

LC3 staining revealed a typical dot-like staining pattern in the cytoplasm of tumour cells. Tumours with no LC3 staining were classified as negative, and tumours demonstrating a dot-like staining, regardless of the amount of stained cells, were classified as positive. Nerves showed positive staining for LC3 and thus served as an internal staining control (Fig. 3). Among the total 128 cases available for LC3 analysis, 88 (68.8 %) were classified as LC3 negative and 35 (27.3 %) as LC3 positive. No statistical correlation was detected between LC3 and p62/Beclin-1 expression. LC3 positivity was significantly associated with a poor differentiation grade (*p* = 0.016; chi-square analysis).

### ULK1 immunostaining

Tumour cell nuclei lacked ULK1 immunostaining. Cases with moderate or strong cytoplasmic staining occasionally also demonstrated a significant membranous staining pattern. Tumour with missing or weak staining were classified as negative (0), and tumours with moderate or strong cytoplasmic and/or membranous staining were classified as positive (+). No statistical correlation was detected between ULK1 staining and survival. ULK1 positive staining was significantly associated with the presence of lymph node metastasis (*p* = 0.037, chi-square analysis). There was no association with the remaining clinicopathological parameters (data not shown).

### p62, LC3, Beclin-1 and ULK1 expression and KRAS mutational status

KRAS mutation analysis of the present cohort was performed in a previous study [21]; thus, the KRAS status of most patients (*n* = 124) was known. Cytoplasmic/nuclear p62 expression was not related to KRAS mutational status (*p* = 0.607). Furthermore, neither cytoplasmic nor nuclear Beclin-1 expression was related to KRAS mutational status. LC3 and ULK1 expression was also not related to KRAS mutational status.

### Prognostic relevance of p62, Beclin-1, LC3 and ULK1 immunoexpression with respect to KRAS mutational status

**Fig. 6:**
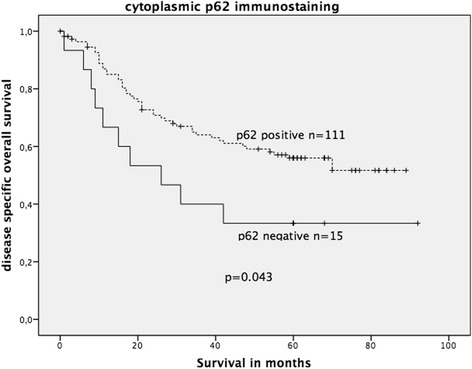
Kaplan-Meier survival plot of 126 colorectal cancers (KRAS wildtype and KRAS mutated) in relation to p62 immunostaining intensity. p62-negative CRC revealed a significantly decreased OS (log-rank test: *p* = 0.043)

Patients with cytoplasmic p62-negative CRC exhibited a significantly shortened OS (*p* = 0.043, Fig. 6). Histological grading, lymph node metastases, tumour stage and distant metastases were also significantly correlated with patient outcome (Table 2). Nuclear p62 immunostaining did not show a statistical significant association with OS.

**Table 2:** Univariate analysis (Kaplan-Meier, log-rank test) for prognostic significance of p62, histological grading, lymph node and distant metastases and tumour stage in relation to tumour-specific overall survival in the complete cohort

Parameter	*p* value
Cytoplasmic p62 (negative/positive)	0.043*
Cytoplasmic Beclin-1 expression (positive/negative)	0.464
Nuclear Beclin-1 expression (positive/negative)	0.106
Histological grading (G1-G3)	0.032*
Lymph node metastases (pN0 vs pN+)	<0.001*
Tumour stage (pT1-4)	<0.001*
Distant metastases (M0 vs M1)	<0.001*

*Statistically signifcant

Cytoplasmic p62 immunostaining and the relevant clinicopathological parameters were subjected to multivariate analyses. Only distant metastases qualified as an independent prognostic marker of OS (*p* = 0.004, Table 3). In Kaplan-Meier survival analysis, there was a trend demonstrating the negative prognostic effect of decreased p62 expression solely in the subgroup of CRC with KRAS mutation (*p* = 0.062, log-rank test) in comparison to the CRC subgroup with wildtype KRAS (*p* = 0.457, log-rank test).

**Table 3:** Results of multivariate analysis (Cox regression) to determine the independent prognostic value of different variables in relation to tumour-specific overall survival in the complete cohort

Covariate	Relative risk	95 % CI	*p* value
p62	0.764	NS	0.619
Histological grading	1.239	NS	0.392
Lymph node metastases	0.794	NS	0.510
Tumour stage	1.647	NS	0.141
Distant metastases	3.176	1.453-6.940	0.004

*NS* statistically not significant

**Fig. 7:**
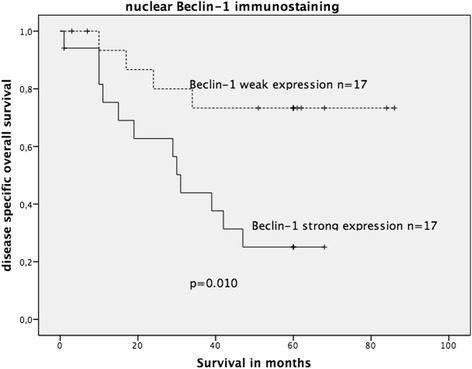
Kaplan-Meier survival plot of 34 KRAS-mutated colorectal cancers in relation to nuclear Beclin-1 immunoexpression. Strong nuclear Beclin-1 expression was significantly associated with decreased OS survival (log-rank test: *p* = 0.010)

In the complete cohort, neither cytoplasmic nor nuclear Beclin-1 expression was associated with OS. When stratified into wildtype and mutated KRAS, positive nuclear (but not cytoplasmic) Beclin-1 expression was significantly associated with an unfavourable OS solely in the mutated KRAS CRC group (*p* = 0.010; log-rank test; Fig. 7), but not in the wildtype KRAS CRC group (figure not shown).

**Fig. 8:**
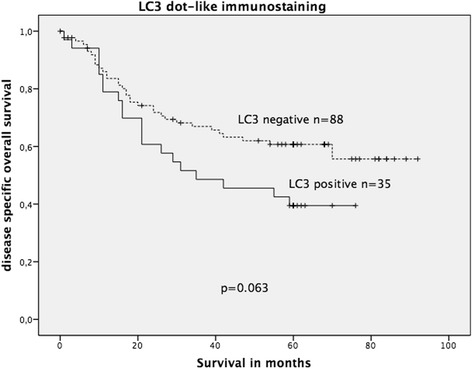
Kaplan-Meier survival plot of 123 colorectal cancers (KRAS wildtype and KRAS mutated) in relation to dot-like LC3 immunostaining intensity. LC3 expression lacked a significant association with OS; however, there was a statistical trend of LC3 expression being associated with decreased OS (log-rank test: *p* = 0.063)

**Fig. 9:**
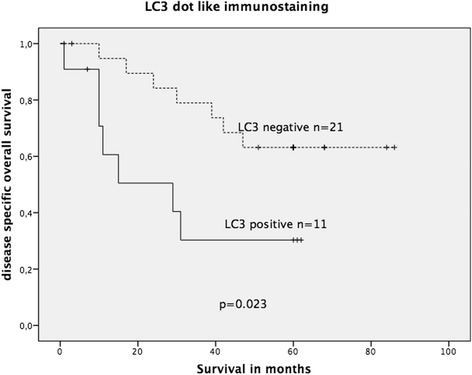
Kaplan-Meier survival plot of 32 KRAS-mutated colorectal cancers in relation to dot-like LC3 immunoexpression. LC3 expression was significantly associated with decreased OS survival (log-rank test: *p* = 0.023)

To evaluate LC3 immunostaining, Kaplan-Meier survival analysis was performed on the complete cohort (KRAS wildtype and KRAS mutated). LC3 expression did not show significant association with OS in the complete cohort; however, a statistical trend was observed (*p* = 0.063, Fig. 8). When stratified into wildtype and mutated KRAS groups, positive LC3 dot-like staining was significantly associated with decreased OS in the mutated KRAS CRC group (log-rank test: *p* = 0.023, Fig. 9) but not in the wildtype KRAS CRC group (*p* = 0.23).

ULK1 expression was not associated with survival neither in the complete cohort nor in the mutated-type CRC.

### Prognostic relevance of p62 and Beclin-1 immunoexpression with respect to UICC stage

**Fig. 10:**
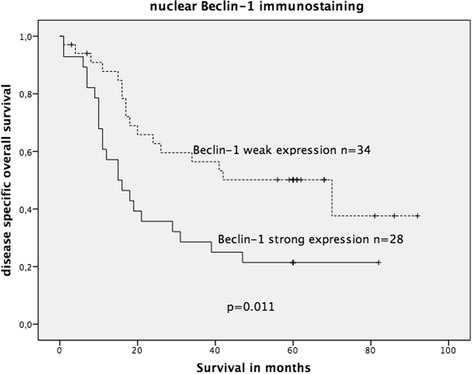
Kaplan-Meier survival plot of 62 colorectal cancers with advanced stage UICC III/IV (all treated with 5-FU-based chemotherapy) in relation to nuclear Beclin-1 immunoexpression. In this subgroup, strong Beclin-1 expression was significantly associated with decreased OS survival (log-rank test: *p* = 0.011)

Survival analysis was performed in early stage UICC I/II CRCs and advanced stage UICC III/IV CRCs. Neither nuclear nor cytoplasmic p62 immunostaining showed a statistically significant association with OS in both subgroups (data not shown). Positive nuclear (but not cytoplasmic) Beclin-1 expression was significantly associated with an unfavourable OS solely in the advanced UICC III/IV CRCs (*p* = 0.011; log-rank test; Fig. 10) but not in early stage UICC I/II CRCs (figure not shown). All patients in this cohort with UICC III/IV CRCs received 5-FU-based chemotherapy.

## Discussion

Autophagy is a cellular pathway that regulates transportation of cytoplasmic macromolecules and organelles to lysosomes for degradation [22] and ensures protein and organelle homeostasis [1, 23]. Cancer cells have a high demand for energy equivalents and specific metabolites, and these resources can be provided by autophagy.

Interestingly, autophagy processes in cancer may show both tumour-promoting and tumour-suppressing effects depending on many factors, such as tissue type, tumour stage and the type of oncogenic mutations involved in the different tumour types [2, 22]. Thus, it is not surprising that immunohistochemical evaluation of autophagy-related markers has revealed different results according to prognosis. However, autophagy inhibitors may serve as a potential therapeutic intervention in some cancers, and thus autophagy may become a clinically relevant focus in cancer diagnosis and therapy.

In this study, we performed immunohistochemically analysis of the autophagy-related markers p62, LC3, Beclin-1 and ULK1 in a cohort of CRCs. We acknowledge that measurement of the autophagy flux is challenging, since autophagy flux is a dynamic, multiple-stage process, including autophagosome formation, maturation and fusion with lysosomes as well as breakdown and release of macromolecules back to the cytosol. Thus, the accumulation of autophagosomes as roughly measured by immunohistochemistry could indicate either autophagic activation or a blockage of downstream actions, such as inefficient fusion or decreased lysosomal degradation [1]. However, immunohistochemical analysis of autophagy-related markers such as LC3 offers the opportunity to generate a temporary picture of the amount of autophagosome formation in tumour tissue at a specific point of time in a certain clinical setting [7].

Autophagy-related markers have been a subject of recent immunohistochemical studies in gastrointestinal adenocarcinomas (cholangiocarcinoma, pancreatic adenocarcinoma, oesophageal adenocarcinoma, gastric adenocarcinoma). Table 4 summarizes previously published study results and gives an overview about the expression of autophagy markers in cancer tissue and their potential clinical relevance in gastrointestinal carcinomas other than CRC.

**Table 4:** Literature review on immunohistochemical expression of autophagy-related proteins in gastrointestinal adenocarcinoma other than colorectal carcinoma

Tumour type	Number	p62	Beclin-1	LC3B	ULK1	Clinical relevance	References
Gastric adenocarcinoma	61	Positive nuclear 57 % Positive cytoplasmic 61 %	–	–	–	p62: High p62 expression associated with low differentiation grade but less lymph node metastasis	[26]
	510	Positive 49.2 %	Positive 24.7 %	Positive 15.5 %	–	Autophagy (defined as at least two of three markers positive) was associated with poor survival	[42]
	75	–	Positive 70.6 %	–	–	No survival analysis performed. Increased Beclin-1 expression in well differentiated gastric cancer compared to normal mucosa. Decreased Beclin-1 expression in poorly differentiated gastric cancer	[43]
Pancreatic adenocarcinoma	18	Positive nuclear 78 % Positive cytoplasmic 56 %	–	–	–	p62: none	[26]
	73		Positive 47.9 %	Positive 83.3 %		Beclin-1 overexpression was associated with poor prognosis	[44]
	63		Positive 22.2 %			Beclin-1 overexpression was associated with favourable prognosis	[45]
Intrahepatic cholangiocarcinoma	108	–	Strong expression 24.1 %, 48,1 % moderate and 27.8 % weak/negative expression	–	–	Low Beclin-1 expression was associated with worse OS and DFS. Low Beclin-1 expression correlated with lymph node metastasis	[46]
Oesophageal adenocarcinoma	116	–	–	High p62 81 % High LC3B 83.6 %	–	Low LC3B and low p62 cytoplasmic expression was associated with worse OS	[33]

The prognostic relevance of LC3 expression in CRC has been addressed in only a few studies so far. One study demonstrated a prognostic impact of LC3 expression in CRC, while another failed to demonstrate prognostic relevance of LC3 expression [24, 25]. Our study showed a statistical trend demonstrating reduced OS in CRC patients with LC3-positive CRC.

Only few studies on p62 and colorectal cancer have been published thus far. In a recent study on 178 colon carcinomas treated with adjuvant 5-FU, Beclin-1 overexpression (but not p62) was significantly associated with worse OS [25]. Another study was performed on p62 expression in various gastrointestinal cancers; however, this study included only 45 colon cancers and thus the results may not be reliable [26]. Immunohistochemical analysis of p62 expression has been examined in various other human malignancies with differing results. High cytoplasmic p62 expression and LC3 overexpression were associated with an unfavourable prognosis in oral squamous cell carcinoma [27, 28]. High cytoplasmic p62 expression was associated with decreased OS in triple negative breast cancer [29] and lung adenocarcinoma [30]. In these studies, increased cytoplasmic p62 expression was accompanied by an unfavourable course of cancer disease. However, in the present study, cytoplasmic negativity was significantly associated with a reduced cancer-specific OS, but did not qualify as an independent prognostic marker in multivariate survival analysis. At first glance, our results seem surprising, since the previously published studies revealed increased and not decreased p62 expression as a negative prognostic marker. On the other hand, there are also diverging reports about the potential prognostic relevance of p62 in human cancer [27]. p62 is a protein, which is embedded in multiple pathways and can exert manifold functions. It is well known that p62 may act as an adaptor protein that transports ubiquitinated protein aggregates to autophagosomes via its association with LC3B. In addition, p62 influences the oxidative stress response, which is regulated by the Keap1-Nrf2 system. However, given the fact that negative (and not increased) p62 cytoplasmic expression is associated with worse OS in our study, there has to be another explanation for this rather surprising finding. Indeed, at least two p62-related mechanisms may explain the aggressive course of p62-negative colorectal cancer:

1) p62 may also act as a tumour suppressor since it is capable of inducing autophagic degradation of regulators of the Wnt signalling pathway [31]. It is tempting to speculate that deficiency of p62 might upregulate this oncogenic pathway resulting in increased biological aggressiveness.

2) Recent studies demonstrated that the lack of cdk1-mediated phosphorylation of p62 leads to enhanced cell proliferation and tumorigenesis in response to RAS-induced transformation [32]. A decrease in p62 protein thus may lead to a faster exit from mitosis, which translates into enhanced cell proliferation. These mechanisms might provide a rationale explanation for the worse prognosis of p62-negative CRC. In addition, this finding might also explain the significant prognostic impact in the KRAS-mutated subgroup due to increased RAS-signalling.

In fact, results of a very recent study on 116 oesophageal adenocarcinomas are in line with our results. The immunohistochemical study of Adams et al. established missing/low (and not increased) p62 immunostaining as a predictor of worse prognosis in this cohort [33].

To complicate the evaluation of autophagy-related proteins, both cytoplasmic and nuclear staining has been described in previous studies. Although autophagy-related markers are thought to exert their effects mainly in the cytoplasm, these markers may be localized both in the cytoplasmic compartment as well as in the nuclear compartment [34]. Indeed, in a previous study on CRCs, nearly half of the cancers exhibited a significant nuclear Beclin-1 staining pattern [16].

Autophagy can exert both tumour-suppressive and tumour-promoting effects in human malignancies [22]. Recent studies showed that activation of oncogenic RAS, which is capable of inducing tumour growth, is sufficient to upregulate basal autophagy [35]. RAS mutation is a frequent and early tumour-promoting event in CRC. We analysed p62 expression in KRAS wildtype and KRAS-mutated-type CRC in this study and investigated the prognostic power of p62 expression. p62 expression was not correlated to RAS mutational status, but the prognostic power of p62 was lost in the wildtype KRAS subgroup, whereas it was observed as a trend (*p* = 0.062) in the KRAS-mutated subgroup. However, these results must be interpreted with caution. Cytoplasmic and nuclear Beclin-1 expression failed to show prognostic power in the complete cohort and especially in the wildtype KRAS CRC subgroup. However, in the mutated KRAS CRC subgroup, increased nuclear Beclin-1 expression was significantly associated with decreased OS.

The altered prognostic effect of autophagy-related markers in KRAS-mutated CRC represents a novel finding of our study. Interestingly, similar to Beclin-1, LC3-positive CRC with mutated KRAS demonstrated a significantly reduced OS but not in the KRAS wildtype CRC group, strengthening this finding. Thus there seems to be an alteration of the autophagy flux in the setting of KRAS mutation. A larger sample size of this subgroup with RAS-mutated cancers should be examined to confirm these results.

A previous study on CRCs reported the presence of nuclear Beclin-1 staining; however, the nuclear staining pattern lacked any prognostic relevance [16]. Nevertheless, the results of the present study need to be reproduced in larger cohorts. It is particularly important to conduct additional studies of autophagy-related markers with a focus on the cellular localizations of the proteins. Taking into account the results of previous studies on the prognostic relevance of p62 expression in CRC, why both underexpression and overexpression of this autophagy-related protein were demonstrated to influence survival in CRC as well as in other malignancies remains unclear. This question cannot be answered by the present study, since this study remains descriptive and lacks functional investigations.

ULK1 expression has been addressed in only one study on CRC so far [36]. Zou et al. were able to demonstrate that increased expression levels of ULK1 predicted poor prognosis in CRC. However, in our study with a smaller number of patients, we were not able to confirm these results. Nevertheless, in our study, increased ULK1 expression was significantly associated with the presence of lymph node metastasis. It has to be critically emphasized that in the study by Zhou et al., level of significance was rather low.

Analysis of autophagy-related proteins in human malignancies represents a valuable area of research due to the potential future therapeutic relevance.

Recent in vitro and in vivo studies in preclinical models suggested that modulation of autophagy can be used as a therapeutic modality to enhance the efficacy of conventional therapies, including chemotherapy and radiation therapy [37–40].

Clinical studies with autophagy inhibition are already being conducted [41] with most of the studies using hydroxychloroquine as an autophagy inhibitor. However, autophagy inhibition in human malignancies remains a topic of clinical studies and is not yet part of established therapeutic approaches. Our data show that p62, LC3 and Beclin-1 represent promising novel therapeutic targets especially in the subgroup of KRAS-mutated colorectal cancer patients.

## Conclusions

Our study showed that decreased cytoplasmic p62 expression was significantly associated with an unfavourable tumour-specific OS, especially in the subgroup of KRAS-mutated CRCs. Nuclear Beclin-1 and dot-like LC3 expression were also associated with decreased OS solely in the mutated KRAS CRC subgroup. ULK1 expression completely lacked prognostic relevance. Thus p62, Beclin-1 and LC3 represent promising markers of novel targeted therapies, especially in the clinically relevant group of advanced CRCs treated by chemotherapy and prognostic unfavourable KRAS-mutated CRCs.

## Abbreviations

AMPK, AMP-activated protein kinase; BRCA1, breast cancer-associated antigen; CRC, colorectal carcinoma; H&E, haematoxylin and eosin; LC3, light chain 3; KRAS, Kirsten RAS; PI3K, phosphoinositide-3 kinase; UICC, Union internationale contre le cancer; ULK1, uncoordinated (UNC) 51-like kinase 1; 5-FU, 5-fluorouracilk

## Additional files

Additional file 1: Positive autophagy staining control. (JPG 545 kb) Additional file 2: Raw data converted from SPSS to Excel. (XLSX 18 kb)
