# Supplementary figures and images for: Prognostic relevance of autophagy-related markers LC3, p62/sequestosome 1, Beclin-1 and ULK1 in colorectal cancer patients with respect to KRAS mutational status
Source: World J Surg Oncol. 2016 Jul 22;14:189. doi: 10.1186/s12957-016-0946-x (PMC4957418; doi:10.1186/s12957-016-0946-x)

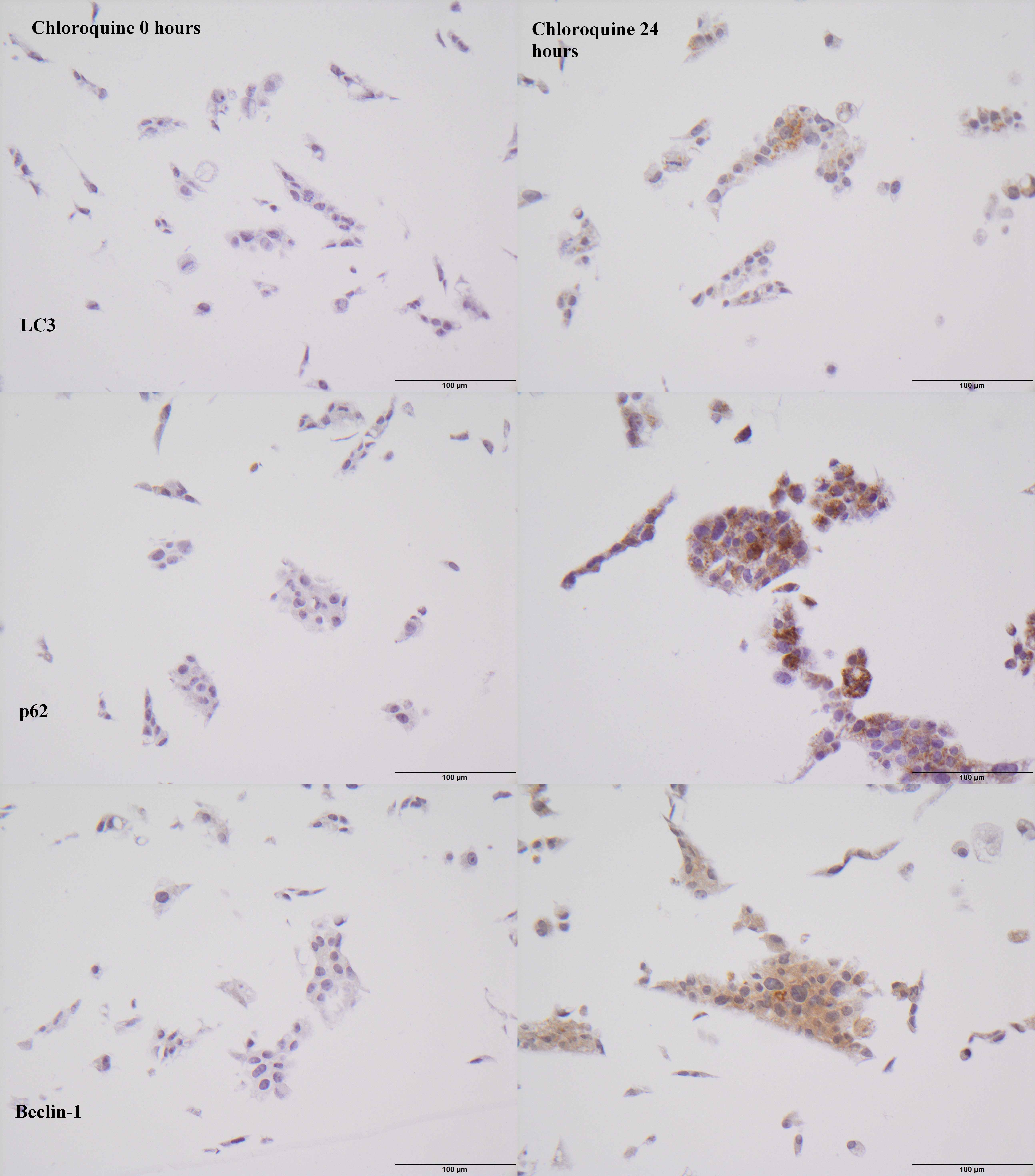

Supplement: Additional file 1: — Positive autophagy staining control. (JPG 545 kb) [file 12957_2016_946_MOESM1_ESM.jpg]
